# Prior high demand is associated with lower subsequent performance in a manual antisaccade task

**DOI:** 10.3389/fpsyg.2026.1882059

**Published:** 2026-08-05

**Authors:** Tianyi Fan, Mingchuan Ma, Lihua Mao

**Affiliations:** School of Psychological and Cognitive Sciences, Peking University, Beijing, China

**Keywords:** accuracy, cognitive control, cue-target interval, manual antisaccade task, post-cue processing, prior cognitive demand, reaction time

## Abstract

Prior high cognitive demand can be followed by lower performance on subsequent cognitive-control tasks, but the nature of this post-demand disadvantage remains unclear. We examined whether later performance differences were selectively Anti-specific, broader across task types, or varied with post-cue preparation time. Eighty-four participants completed an online sequential-task experiment. Task 1 was an adaptive block-counting task that manipulated prior performance demand. Task 2 was a keyboard-response manual antisaccade task with Pro and Anti conditions and cue-target intervals (CTIs) of 50, 200, and 400 ms. Accuracy and correct-trial reaction time (RT) were analyzed. The high-demand Task 1 version produced lower accuracy than the low-demand version. In Task 2, the high-demand group had lower accuracy than the low-demand group. The simple interaction analysis revealed that the Anti Group × CTI interaction was significant, whereas the Pro interaction was not. Group differences were present at all three Anti CTIs and were numerically largest at 400 ms. Neither corrected RT simple interaction was significant, so no downstream RT simple effects were interpreted. Prior high demand was associated with lower subsequent manual antisaccade accuracy. The Anti CTI pattern is consistent with, but does not establish, differences in post-cue processing. Because conditions used separate task links, the results are associations rather than definitive causal estimates.

## Introduction

Prior high cognitive demand can be followed by poorer performance on a subsequent task, but the interpretation of this sequential-task effect remains debated. Early accounts often framed such effects as evidence for a temporary depletion of self-control resources. However, large preregistered and multisite studies have challenged strong and uniform depletion interpretations (Dang et al., 2021; Lurquin and Miyake, 2017; Vohs et al., 2021). Recent reviews instead suggest that sequential-task effects may arise from multiple changes in later task processing, including changes in attention, motivation, control allocation, and time-on-task-related fatigue (Forestier et al., 2022; Mangin et al., 2022). The present study therefore asked how prior high demand is associated with later performance in an inhibition-relevant but componentially complex task.

One common interpretation is that prior self-control or high-demand tasks impair later inhibition (Dang et al., 2017). This view is plausible because many outcome tasks require participants to suppress a dominant or cue-driven response. However, poorer performance on an inhibition-relevant task does not necessarily isolate inhibition as the impaired process. In the antisaccade literature, poorer performance has been linked to goal activation, goal maintenance, attentional consistency, cue-to-target translation, and perceptual identification, rather than inhibition alone (Meier et al., 2018; Unsworth et al., 2004, 2022). Similarly, process-oriented work in the sequential-task literature suggests that prior high demand can alter later decision processes without necessarily producing a selective inhibition deficit (Lin et al., 2020). The present study therefore avoided treating an Anti-trial disadvantage as a process-pure inhibition measure and instead examined whether the pattern was selectively Anti-specific or reflected broader post-cue processing changes.

The present design required a prior-demand manipulation that was demanding but structurally distinct from the later manual antisaccade task. We therefore used an adaptive block-counting task as Task 1. In the low-demand version, participants reported the total number of blocks in a sequence. In the high-demand version, participants reported the numbers of small and large blocks separately. The high-demand version was intended to increase sustained updating, monitoring of size-category changes, maintenance of counting goals, and response-complexity demands, while keeping the general stimulus context comparable across groups. Task 1 accuracy was used as a performance-demand check, not as a direct measure of depletion or as a process-pure index of cognitive demand.

A key theoretical question is how a prior counting/updating task might affect later manual antisaccade performance. We propose a cautious carryover account: sustained updating and monitoring in Task 1 may reduce attentional stability or control allocation in the subsequent task, increase attentional lapses, or produce residual task-set interference. Such changes could make cue encoding, cue-location maintenance, cue-response translation, stimulus–response binding, and rule implementation less reliable in Task 2. The present design cannot distinguish these candidate mechanisms, but it can test whether the later performance difference is selectively Anti-specific or is better described as a broader post-cue performance disadvantage.

Task 2 was a keyboard-response version of the manual antisaccade task. This task is inhibition-relevant because the Anti condition requires participants to respond to the target opposite the cue rather than to the cue-compatible location. However, manual antisaccade performance is not inhibition-pure. Frischkorn and Oberauer (2025) argued that performance reflects multiple component processes, including inhibition of the cue-driven response, cue-to-target translation, stimulus–response bindings in procedural working memory, response preparation and execution, and target identification. Related computational work also shows that antisaccade errors cannot be reduced to early inhibition failures alone (Aponte et al., 2018). Thus, the manual antisaccade task is useful for examining inhibition-relevant performance, but the present results should not be interpreted as isolating inhibition as a single causal process.

The present design further varied cue-target interval (CTI). The three CTIs of 50, 200, and 400 ms were selected before data analysis to maintain comparability with the manual antisaccade paradigm of Frischkorn and Oberauer (2025), from which the present Task 2 was adapted. In that paradigm, the delay between the cue and target was varied across the same three levels to examine the time course or speed of task-relevant post-cue processes. We retained these values because they allowed the present task to remain close to the source paradigm while sampling relatively early, intermediate, and later post-cue processing windows. Conceptually, a 50-ms CTI leaves minimal time for post-cue preparation, whereas 200- and 400-ms CTIs provide progressively more time for cue encoding, cue-location maintenance, cue-response translation, stimulus–response binding, and rule implementation (Adam et al., 2015; Pierce and McDowell, 2016; Van Gerven et al., 2016). CTI should not be treated as a pure index of inhibition or as a direct marker of any single mechanism; rather, it provides temporal leverage over multiple post-cue processes (Aponte et al., 2018; Frischkorn and Oberauer, 2025).

The study tested three behavioral interpretations of the later-task performance difference. A selectively Anti-specific account would predict a disproportionately larger high-demand disadvantage in the Anti condition than in the Pro condition. A broader performance-disadvantage account would predict lower performance following high- than low-demand Task 1 across both task types. A post-cue processing account would predict that the size of the group difference varies with CTI, especially in the Anti condition where the cue location must be translated into the opposite response location. These alternatives were evaluated using accuracy as the primary outcome and correct-trial RT as the secondary behavioral outcome.

## Materials and methods

### Participants

Participants were recruited through the Credamo online platform and completed the experiment online in a web browser. Before participation, all participants provided informed consent electronically and completed a demographic questionnaire. The experiment was implemented in PsychoPy and administered online.

A total of 84 participants were included in the final analyses, with 43 in the low-demand group and 41 in the high-demand group. Sample characteristics and the design overview are summarized in Table 1. Participants were recruited from the same Credamo marketplace participant pool. The low- and high-demand conditions were implemented through two condition-specific task links that used the same eligibility criteria and compensation. No single-link randomization, matching, or stratified allocation procedure was implemented. Therefore, the design supports a comparison of groups completing different demand conditions, but it cannot fully distinguish demand-related effects from unobserved between-link compositional differences.

**Table 1:** Sample characteristics and design overview.

Variable	Full sample	Low-demand group	High-demand group
*n* analyzed	84	43	41
Age, M (SD)	29.5 (8.37)	30.8 (10.27)	28.1 (5.58)
Age range, years	20–59	20–59	20–46
Men, *n* (%)	36 (43%)	21 (49%)	15 (37%)
Women, *n* (%)	48 (57%)	22 (51%)	26 (63%)
Task 1 condition	Adaptive block-counting task	One total count	Separate counts for small and large blocks
Task 2	Manual antisaccade task with CTIs of 50, 200, and 400 ms	—	—

Because the study did not include a formal *a priori* power calculation before data collection, we report an achieved-*N* sensitivity analysis for the Group × Task type × CTI interaction. As a defensible approximation, the mixed-design interaction was treated as an *F* test with numerator df = 2 and denominator df = 164. With *N* = 84, alpha = 0.05, and target power = 0.80, the detectable effect was approximately Cohen’s *f* = 0.342, corresponding to partial eta squared = 0.105. This sensitivity analysis is approximate because it does not incorporate the exact repeated-measures covariance or sphericity structure, and it is reported as a transparent sample-size sensitivity check rather than as an *a priori* justification.

The study was approved by the Committee for Protecting Human and Animal Subjects at the School of Psychological and Cognitive Sciences of Peking University, China. Participants provided informed consent electronically before participation. The study was conducted in accordance with the Declaration of Helsinki.

### Design

The study used a 2 × 2 × 3 mixed design. Group was a between-subjects factor with two levels: low-demand and high-demand. Task type and CTI were within-subject factors. Task type had two levels, Pro and Anti, and CTI had three levels, 50, 200, and 400 ms. The primary behavioral outcome was accuracy. Correct-trial reaction time was analyzed as a secondary behavioral outcome.

### Procedure overview

**Figure 1:**
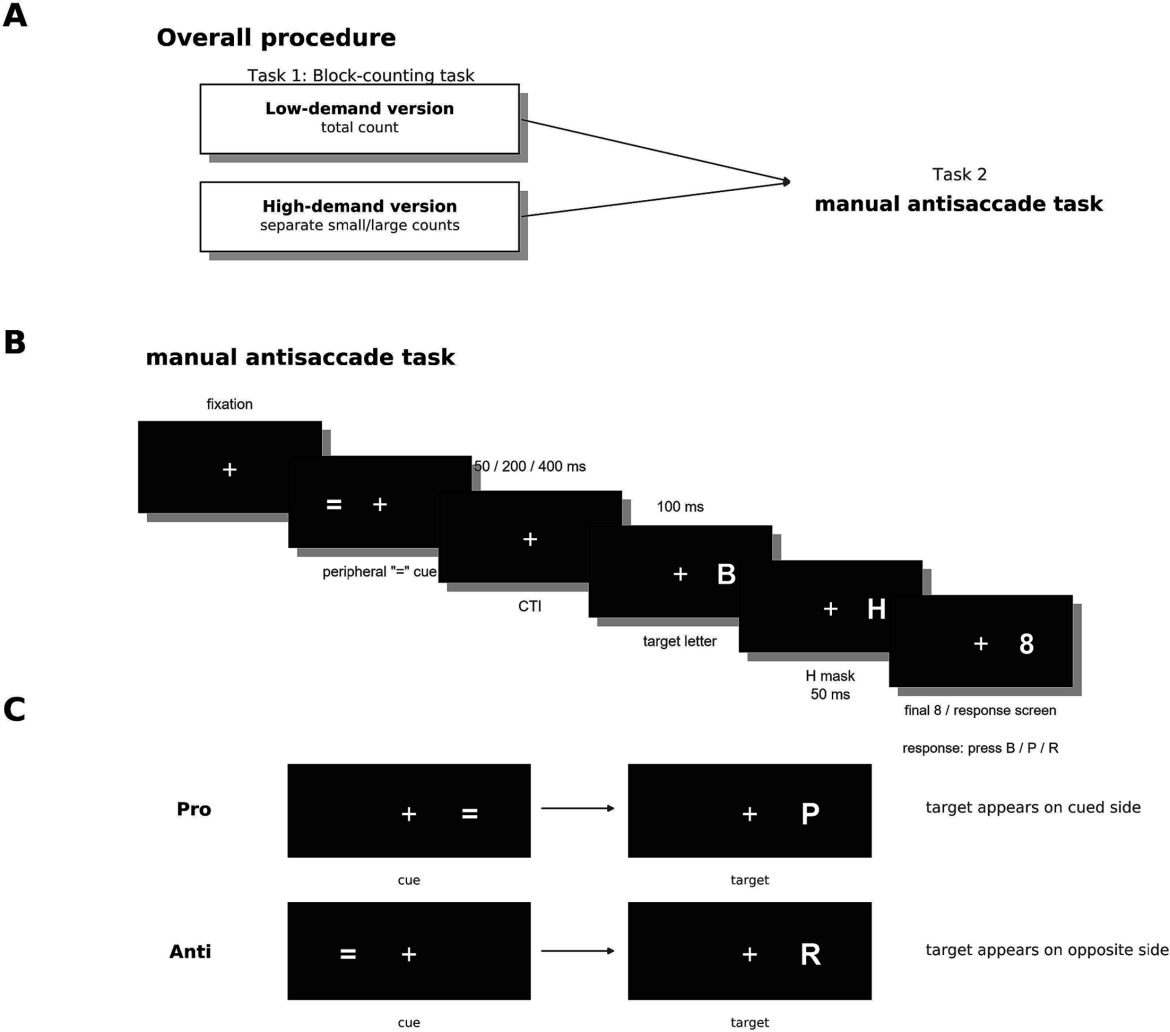
Experimental procedure and Task 2 trial structure. Panel **(A)** shows the overall sequential-task procedure. Participants completed either the low-demand or high-demand version of Task 1, followed by the manual antisaccade task. Panel **(B)** illustrates the Task 2 trial structure. Each trial included a fixation display, a peripheral equals-sign cue, a cue-target interval (CTI) of 50, 200, or 400 ms, a briefly presented target letter, masks, and a keyboard response. Panel **(C)** illustrates the Pro and Anti spatial mappings: in Pro trials, the target appeared on the cued side; in Anti trials, the target appeared on the opposite side.

The experiment followed a two-stage sequential-task structure. Participants first completed Task 1, an adaptive block-counting task designed to manipulate prior performance demand. They then completed Task 2, a keyboard-response version of the manual antisaccade task, adapted from Frischkorn and Oberauer (2025). Figure 1 illustrates the overall procedure and the Task 2 trial structure.

### Task 1: adaptive block-counting task

Task 1 was an adaptive block-counting task. On each trial, participants viewed a sequence of small and large blocks and then entered the required count. In the low-demand group, participants reported the total number of blocks in the sequence. In the high-demand group, participants reported the numbers of small and large blocks separately. The response interface and scoring rule were matched to the assigned condition: low-demand trials required one total-count response, whereas high-demand trials required two category-specific responses. A trial was scored as correct only when the submitted count or counts exactly matched the required answer.

The high-demand version was intended to impose greater performance demand through category-specific updating, continuous monitoring of block-size changes, maintenance of counting goals, and more complex response entry and scoring. Because the counting task was not itself an antisaccade or inhibition task, it reduced structural overlap with Task 2 and allowed us to examine downstream effects on later inhibition-relevant and cue/rule-guided performance.

Participants first completed two practice trials with feedback. The formal phase was programmed to last up to 15 min and ended when either 90 formal trials had been completed or the time limit was reached. No trial-by-trial feedback was provided during the formal phase. Task difficulty was adjusted across formal trials according to previous-trial accuracy by changing the total number of blocks, the switching intensity between block sizes, and the presentation duration of each block. The presentation duration of each block ranged from 0.1 to 0.8 s. Full adaptive difficulty rules are provided in the Supplementary Methods, and the task structure is summarized in Supplementary Figure S2.

Overall formal Task 1 accuracy was used to verify that the high-demand version imposed greater performance demand than the low-demand version.

### Task 2: manual antisaccade task

Task 2 was a keyboard-response version of the manual antisaccade task. Participants completed one Pro block and one Anti block. The order of the Pro and Anti blocks was assigned according to participant number parity. Each block began with 10 practice trials with feedback, followed by 180 formal trials without feedback. The formal task included 360 trials in total, with 180 Pro trials and 180 Anti trials. Each task type × CTI cell included 60 trials. Target letters and spatial locations were balanced across CTI levels within each task type.

Each trial began with a central fixation, followed by a peripheral equals-sign cue. After a CTI of 50, 200, or 400 ms, a target letter (B, P, or R) was presented for 100 ms and then followed by brief visual masks. Participants identified the target letter by pressing the corresponding B, P, or R key. In Pro trials, the target appeared on the same side as the cue. In Anti trials, the target appeared on the side opposite the cue. Thus, Anti trials required participants to apply the opposite-location rule before identifying the target letter. Trials within each block were presented in randomized order.

The three CTIs were chosen before data analysis by following the manual antisaccade paradigm of Frischkorn and Oberauer (2025), from which the present keyboard-response task was adapted. Retaining 50-, 200-, and 400-ms CTIs allowed the present task to remain comparable with that source paradigm while sampling short, intermediate, and longer cue-target intervals. The 50-ms interval provided minimal post-cue preparation time, whereas the 200- and 400-ms intervals provided progressively more time for cue encoding, cue-location maintenance, cue-response translation, stimulus–response binding, and rule implementation. These CTIs were therefore used to obtain temporal leverage over post-cue processing rather than to isolate a single process.

### Timing and transition information

Task 1 consisted of two practice trials followed by an adaptive formal phase. The formal phase was programmed to end after either 90 formal trials or 15 min, whichever occurred first. All included participants completed fewer than 90 formal trials, indicating that the programmed time limit rather than the trial limit ended the formal phase. The mean number of completed formal Task 1 trials was 72.77 (SD = 5.72) in the low-demand group and 45.63 (SD = 6.30) in the high-demand group. After Task 1, participants advanced through a participant-paced transition instruction screen before beginning Task 2; no fixed rest interval was imposed. Task 2 contained one Pro block and one Anti block, with block order determined by participant-number parity. Each block included 10 practice trials and 180 formal trials, yielding 360 formal trials total and 60 trials per task type × CTI cell. Trial timing included a variable fixation interval, the CTI, a 100-ms target, brief masks, and a response-dependent ending followed by an inter-trial interval. The deployed raw CSVs contain trial-level RTs and task variables, but do not contain global routine timestamps sufficient to reconstruct exact total participant-level Task 1 duration, the full interval from Task 1 completion to the first Task 2 formal trial, or total Task 2 completion time beyond the programmed timing information reported here.

### Behavioral preprocessing

Practice trials were excluded. Accuracy was calculated from all valid formal trials within each participant × task type × CTI cell, whereas reaction time was calculated from correct trials only. Reaction times were restricted to the range of 150–1,500 ms. After this range-based trimming, reaction-time outliers were removed separately within each participant × task type × CTI cell using a 2.5-SD rule. Trials with missing responses were treated as incorrect for accuracy analyses and were excluded from reaction-time analyses.

No participants were excluded from the main analyses on the basis of Task 2 performance. Sensitivity analyses excluding near-chance or lowest-performing participants were conducted as robustness checks and are summarized in the Results.

### Statistical analysis

Analyses were conducted in R. Statistical significance was evaluated at alpha = 0.05. The primary Accuracy and correct-trial RT analyses used the existing Type III 2 × 2 × 3 mixed ANOVAs, with prior-demand group as a between-subjects factor and task type and CTI as within-subject factors.

Pro- and Anti-specific Group × CTI simple interactions were recalculated using the pooled CTI and Task type × CTI error sources. The two simple interactions were evaluated at alpha = 0.025 (0.05/2). Only the significant Accuracy Anti branch was decomposed further. The three Group-at-CTI and two CTI-within-group simple–simple main effects were evaluated at alpha = 0.01 (0.05/5). No *post hoc* adjustment was used for the two-level Group effects; three-level CTI comparisons were Holm-adjusted within each group. RT used the same hierarchical-testing logic, and no downstream RT tests were conducted because neither simple interaction was significant. Pooled sums of squares, corrected degrees of freedom, pooled error mean squares, *F* values, *p* values, partial eta squared, and decisions are reported in Supplementary Tables S2–S10, S14. The omnibus Accuracy and RT results are summarized in Table 3, and cell-level descriptive statistics are provided in Table 4.

**Table 3:** Mixed ANOVA summary for behavioral outcomes.

Outcome	Effect	*F*	df	*p*	Partial eta squared
Accuracy	Group	153.71	1, 82	<0.001	0.652
Accuracy	Task type	233.10	1, 82	<0.001	0.74
Accuracy	Group × task type	2.18	1, 82	0.144	0.026
Accuracy	CTI	357.91	1.67, 136.62	<0.001	0.814
Accuracy	Group × CTI	0.53	1.67, 136.62	0.558	0.006
Accuracy	Task type × CTI	161.26	1.83, 149.71	<0.001	0.663
Accuracy	Group × task type × CTI	6.22	1.83, 149.71	0.003	0.07
Reaction time	Group	0.14	1, 82	0.706	0.002
Reaction time	task type	7.34	1, 82	0.008	0.082
Reaction time	Group × task type	0.26	1, 82	0.610	0.003
Reaction time	CTI	99.56	1.55, 127.31	<0.001	0.548
Reaction time	Group × CTI	1.53	1.55, 127.31	0.223	0.018
Reaction time	task type × CTI	17.00	1.93, 158.66	<0.001	0.172
Reaction time	Group × task type × CTI	5.84	1.93, 158.66	0.004	0.067

**Table 4:** Descriptive statistics for central Task 2 outcomes.

Group	Task type	CTI (ms)	Accuracy M (SD)	Correct-trial RT M (SD), ms
Low-demand	Pro	50	0.532 (0.103)	686.4 (144.5)
High-demand	Pro	50	0.334 (0.062)	670.2 (144.9)
Low-demand	Pro	200	0.749 (0.110)	558.5 (120.9)
High-demand	Pro	200	0.536 (0.113)	600.6 (137.4)
Low-demand	Pro	400	0.907 (0.097)	500.9 (131.6)
High-demand	Pro	400	0.735 (0.155)	528.4 (128.9)
Low-demand	Anti	50	0.550 (0.120)	671.7 (135.9)
High-demand	Anti	50	0.340 (0.059)	681.4 (204.0)
Low-demand	Anti	200	0.544 (0.124)	640.7 (132.2)
High-demand	Anti	200	0.350 (0.054)	610.6 (180.8)
Low-demand	Anti	400	0.672 (0.161)	586.4 (127.9)
High-demand	Anti	400	0.404 (0.117)	611.4 (189.1)

Accuracy and correct-trial RT values are reported as M (SD) across participant-level cells.

Chance-level tests, performance-exclusion sensitivity analyses, Windows-only analyses, OS-covariate trial-level models where estimable, and frame-rate checks were used as robustness and technical-sensitivity checks. These analyses were interpreted alongside the primary mixed-design results rather than as replacements for the main participant-level analyses.

## Results

### Task 1 performance-demand check

Overall formal Task 1 accuracy was used to verify that the high-demand version imposed greater performance demand than the low-demand version. The low-demand group showed higher Task 1 accuracy than the high-demand group, M = 0.817, SD = 0.115, versus M = 0.660, SD = 0.120, Welch *t*(81.4) = 6.13, *p* < 0.001, Cohen’s *d* = 1.34. The corresponding group difference is shown in Supplementary Figure S1. This result supports the intended performance-demand manipulation while avoiding the stronger interpretation that Task 1 accuracy directly indexes a specific depletion state. Task 1 descriptive statistics are summarized in Table 2.

**Table 2:** Task 1 performance-demand check.

Group	Task 1 accuracy M	SD	*n*	SE	95% CI
Low-demand	0.817	0.115	43	0.018	[0.783, 0.852]
High-demand	0.660	0.120	41	0.019	[0.624, 0.697]

Group comparison: Welch *t*(81.4) = 6.13, *p* < 0.001, Cohen’s *d* = 1.34. The comparison is low-demand minus high-demand.

### Accuracy in the manual antisaccade task

Accuracy showed a significant main effect of Group, *F*(1, 82) = 153.71, *p* < 0.001, partial eta squared = 0.652; accuracy was lower in the high-demand group (M = 0.450, SD = 0.061) than in the low-demand group (M = 0.659, SD = 0.091). The Task type main effect was significant, *F*(1, 82) = 233.10, *p* < 0.001, partial eta squared = 0.740; accuracy was higher for Pro trials (M = 0.634, SD = 0.131) than Anti trials (M = 0.479, SD = 0.146). The CTI main effect was significant, *F*(1.67, 136.62) = 357.91, p < 0.001, partial eta squared = 0.814; accuracy increased from 50 ms (M = 0.441, SD = 0.129) to 200 ms (M = 0.547, SD = 0.131) and 400 ms (M = 0.682, SD = 0.156), and all three Holm-adjusted pairwise comparisons were *p* < 0.001. The Task type × CTI interaction was significant, *F*(1.83, 149.71) = 161.26, *p* < 0.001, partial eta squared = 0.663. The Group × Task type interaction, *F*(1, 82) = 2.18, *p* = 0.144, partial eta squared = 0.026, and Group × CTI interaction, *F*(1.67, 136.62) = 0.53, *p* = 0.558, partial eta squared = 0.006, were not significant. The Group × Task type × CTI interaction was significant, *F*(1.83, 149.71) = 6.22, *p* = 0.003, partial eta squared = 0.070.

The Pro Group × CTI simple interaction was not significant, *F*(1.63, 286.33) = 1.24, *p* = 0.286, partial eta squared = 0.009. The Anti Group × CTI simple interaction was significant, *F*(1.70, 286.33) = 4.32, *p* = 0.019, partial eta squared = 0.029.

At 50 ms, accuracy was higher in the low-demand group (M = 0.550, SD = 0.120) than in the high-demand group (M = 0.340, SD = 0.059), *F*(1, 450.33) = 68.51, *p* < 0.001, partial eta squared = 0.132. At 200 ms, accuracy was higher in the low-demand group (M = 0.544, SD = 0.124) than in the high-demand group (M = 0.350, SD = 0.054), *F*(1, 450.33) = 58.52, *p* < 0.001, partial eta squared = 0.115. At 400 ms, accuracy was higher in the low-demand group (M = 0.672, SD = 0.161) than in the high-demand group (M = 0.404, SD = 0.117), *F*(1, 450.33) = 111.34, *p* < 0.001, partial eta squared = 0.198.

**Figure 2:**
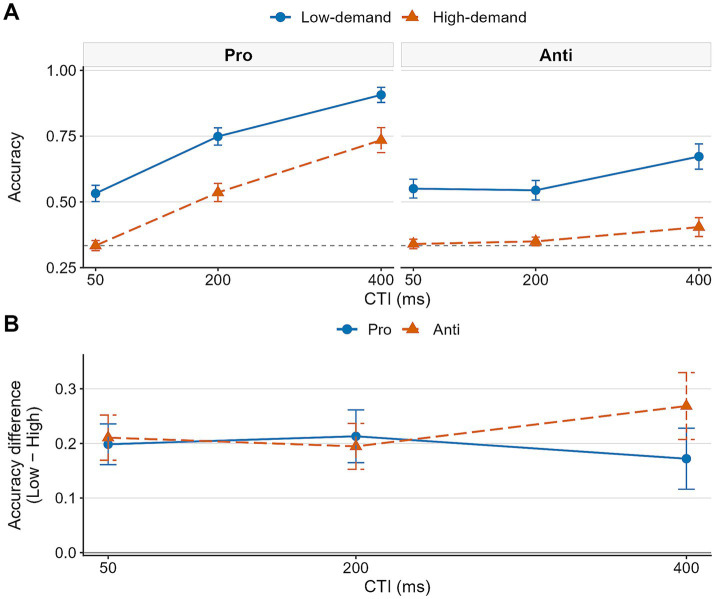
Accuracy in the manual antisaccade task. Panel **(A)** shows mean accuracy as a function of group, task type, and cue-target interval (CTI). The dashed horizontal line indicates nominal chance performance (0.333). Panel **(B)** shows the low-demand minus high-demand accuracy difference across CTIs separately for the Pro and Anti conditions. Error bars represent 95% confidence intervals.

The Anti CTI effect was significant in the low-demand group, *F*(1.89, 286.33) = 30.71, *p* < 0.001, partial eta squared = 0.177: 400-ms accuracy exceeded both 50-ms and 200-ms accuracy (both Holm *p* < 0.001), whereas 50 and 200 ms did not differ (*p* = 0.699). It was also significant in the high-demand group, *F*(1.39, 286.33) = 6.70, *p* = 0.0047, partial eta squared = 0.045: 400-ms accuracy exceeded both 50-ms and 200-ms accuracy (both Holm *p* = 0.008), whereas 50 and 200 ms did not differ (*p* = 0.334). Group differences were present at all three Anti CTIs and were numerically largest at 400 ms. The corresponding accuracy patterns are shown in Figure 2.

Technical sensitivity checks were added because the group-comparability table indicated an operating-system imbalance across condition links (Supplementary Table S1). The low-demand group contained 43 Win32 participants and no non-Win32 participants, whereas the high-demand group contained 34 Win32 and 7 non-Win32 participants. In the Windows-only subset (*N* = 77; low = 43, high = 34), the accuracy group term remained significant (*F* = 138.521, *p* < 0.001), and the Group × Task type × CTI term remained significant (*F* = 5.905, *p* = 0.003). The trial-level accuracy model with OS-binary as a covariate converged without warnings; the OS-binary coefficient was not reliable (*p* = 0.736), whereas the high-demand group coefficient remained reliable (*p* < 0.001). Recorded frame rate did not differ by group in the participant-level check (Welch *p* = 0.795). Browser/user-agent information was not recorded in the available analysis dataset. These checks suggest that the primary pattern was similar in the available technical sensitivity analyses, but they cannot eliminate all possible unobserved between-link differences.

### Floor-effect diagnostics and robustness checks

As a floor-effect diagnostic, we tested whether accuracy in each Group × Task type × CTI cell exceeded the three-alternative chance level (0.333). After Holm correction, the high-demand Pro 50-ms cell and the high-demand Anti 50-ms and 200-ms cells were not reliably above chance, whereas the high-demand Anti 400-ms cell and all remaining cells exceeded chance. These results indicate that some high-demand short-CTI cells were close to floor, so the primary ANOVA should be interpreted together with robustness checks.

Sensitivity analyses excluding one participant with overall Task 2 accuracy <= 0.35 and, alternatively, excluding the bottom 5% of performers preserved the direction of the group effect and the significant Group × Task type × CTI interaction. In the <= 0.35 exclusion analysis, the group effect remained significant, *F*(1, 81) = 149.63, *p* < 0.001, and the three-way interaction remained significant, *F*(1.83, 147.99) = 6.21, *p* = 0.003. In the bottom-5% exclusion analysis, the group effect remained significant, *F*(1, 77) = 130.94, *p* < 0.001, and the three-way interaction remained significant, *F*(1.82, 140.52) = 6.09, *p* = 0.004. Thus, the main low-demand advantage and the Anti-condition CTI pattern were not eliminated by removing near-chance or lowest-performing participants.

Trial-level logistic mixed models were also examined as robustness checks. The full trial-level model showed a strong Group effect, chi-square(1) = 74.22, *p* < 0.001, a CTI effect, chi-square(2) = 830.21, *p* < 0.001, a Group × CTI interaction, chi-square(2) = 21.81, *p* < 0.001, and a Task type × CTI interaction, chi-square(2) = 324.42, *p* < 0.001. The omnibus Group × Task type × CTI interaction was not significant in the full model, chi-square(2) = 4.29, *p* = 0.117. For Anti trials, significant effects of Group, chi-square(1) = 77.81, *p* < 0.001, CTI, chi-square(2) = 116.25, *p* < 0.001, and Group × CTI, chi-square(2) = 17.51, p < 0.001, provided partial robustness support for the group disadvantage and Anti-condition CTI pattern.

### Correct-trial reaction time

**Figure 3:**
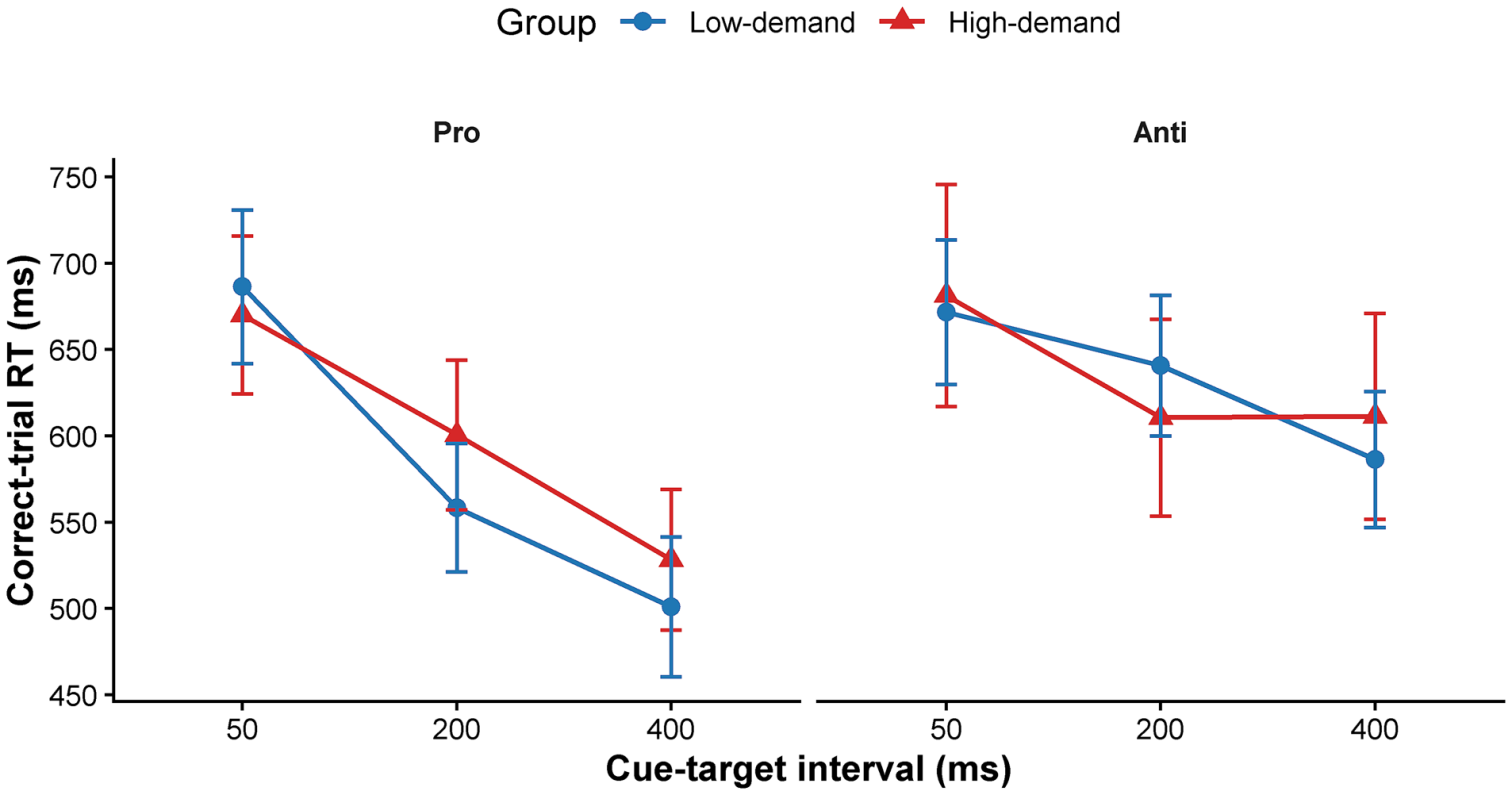
Correct-trial reaction time in Task 2 as a function of cue-target interval (CTI), task type, and prior-demand group. Points show participant-level cell means and error bars show 95% confidence intervals. RTs were computed from correct trials after the manuscript preprocessing rules: 150–1,500 ms range restriction followed by participant × task type × CTI 2.5-SD trimming.

Correct-trial RT showed a significant Task type main effect, *F*(1, 82) = 7.34, *p* = 0.008, partial eta squared = 0.082; RT was shorter for Pro trials (M = 590.61 ms, SD = 121.42) than Anti trials (M = 633.69 ms, SD = 150.62). The CTI main effect was significant, *F*(1.55, 127.31) = 99.56, p < 0.001, partial eta squared = 0.548; RT decreased from 50 ms (M = 677.46 ms, SD = 140.56) to 200 ms (M = 602.54 ms, SD = 114.27) and 400 ms (M = 556.45 ms, SD = 118.28), with all three Holm-adjusted comparisons *p* < 0.001. The Task type × CTI interaction was significant, *F*(1.93, 158.66) = 17.00, *p* < 0.001, partial eta squared = 0.172. The Group main effect, *F*(1, 82) = 0.14, *p* = 0.706, partial eta squared = 0.002, Group × Task type interaction, *F*(1, 82) = 0.26, *p* = 0.610, partial eta squared = 0.003, and Group × CTI interaction, *F*(1.55, 127.31) = 1.53, *p* = 0.223, partial eta squared = 0.018, were not significant. The Group × Task type × CTI interaction was significant, *F*(1.93, 158.66) = 5.84, *p* = 0.004, partial eta squared = 0.067. Neither task-specific Group × CTI simple interaction was significant: Pro, *F*(1.35, 285.97) = 3.11, *p* = 0.066, partial eta squared = 0.021; Anti, *F*(1.98, 285.97) = 2.73, *p* = 0.067, partial eta squared = 0.019. The corresponding RT patterns are shown in Figure 3. Therefore, no downstream RT simple-simple main effects were conducted.

The revised supplementary workbook contains Supplementary Tables S1–S16, covering group comparability, Accuracy and RT omnibus and task-specific follow-ups, descriptives, recorded OS/platform and frame-rate checks, the hierarchical-testing and familywise statistical note, and recorded-platform subset Accuracy and RT analyses.

## Discussion

The present study examined how prior high demand was associated with subsequent performance in a manual antisaccade task. The high-demand group showed lower Task 2 accuracy than the low-demand group. The corrected Anti Group × CTI interaction remained significant, and Group differences occurred at all three Anti CTIs, with the difference numerically largest at 400 ms. The Pro Accuracy interaction and both task-specific RT interactions were not significant. Thus, the behavioral pattern is consistent with, but does not establish, differences in post-cue processing.

The findings should be interpreted cautiously with respect to inhibition. The manual antisaccade task is inhibition-relevant but not inhibition-pure: Anti trials also require cue-location encoding, task-goal maintenance, cue-to-response translation, stimulus–response binding, perceptual identification, and response execution. The results therefore do not isolate inhibition as a causal process. They show a broad accuracy disadvantage associated with prior high demand together with CTI-dependent variation in the Anti condition.

The high-demand counting task required sustained updating of two category-specific counts, monitoring of size changes, goal maintenance, and more complex response entry and scoring. These demands could plausibly influence later cue-guided performance, but the present design cannot identify which process, if any, produced the observed association. The Anti CTI pattern should therefore be treated as descriptive evidence about when the group difference was expressed, not as proof of a specific mechanism.

The RT results did not provide a stable group-related pattern parallel to Accuracy. Although the omnibus three-way interaction was significant, neither task-specific Group × CTI simple interaction was significant, so no downstream RT simple effects were interpreted. This result does not identify the precise cognitive process responsible for the accuracy difference and does not establish a speed-accuracy explanation.

Several limitations should be noted. First, the design compared high-demand and low-demand Task 1 groups but did not include a no-Task-1 baseline. The present results therefore show lower Task 2 performance following high-demand than low-demand Task 1, but they cannot determine whether high demand impaired absolute performance, low demand preserved or facilitated performance, or both Task 1 conditions altered Task 2 performance to different degrees. Second, some high-demand short-CTI cells were close to chance. Sensitivity analyses indicated that the main group disadvantage and Anti-condition CTI pattern were not eliminated by excluding near-chance or lowest-performing participants, but the exact interaction pattern should still be interpreted cautiously. Third, the experiment was conducted online, which may introduce variability in display timing and response recording. Fourth, the task used keyboard responses rather than eye tracking, so the findings speak to manual antisaccade performance rather than directly identifying oculomotor inhibition failures. Finally, CTI provides temporal leverage over post-cue processing but does not isolate a single cognitive or neural mechanism.

The OS imbalance provides an additional technical limitation. Windows-only, OS-covariate, and frame-rate checks were added and did not overturn the main pattern in the available data, but these checks cannot exclude all unmeasured device, browser, display, or recruitment-flow differences. This limitation should be considered when interpreting the group effect.

A key limitation is that the low-demand and high-demand conditions were implemented through condition-specific task links within the same Credamo marketplace participant pool. Because assignment used condition-specific task links rather than a single randomized link, the design did not include single-link randomization, matching, or stratified allocation. Therefore, unmeasured pre-existing differences between condition links cannot be excluded. The present findings should accordingly be interpreted as associations between prior-demand condition and later task performance rather than as fully identified causal estimates.

In conclusion, prior high demand was associated with lower subsequent accuracy in a manual antisaccade task relative to a low-demand comparison condition. The corrected Anti Group × CTI interaction remained significant, with Group differences at all three Anti CTIs and the largest numerical difference at 400 ms, whereas the Pro Accuracy interaction and both task-specific RT interactions were not significant. This pattern is consistent with, but does not establish, differences in post-cue processing. Because the conditions used separate task links, the findings should be interpreted as associations rather than definitive causal estimates.

## Data Availability

The raw data supporting the conclusions of this article will be made available by the authors, without undue reservation.

## References

[ref1] Adam J. J. Jennings S. Bovend'Eerdt T. J. H. Hurks P. P. M. Van Gerven P. W. M. (2015). Switch hands! Mapping temporal dynamics of proactive manual control with anticues. Acta Psychol. 161, 137–144. doi: 10.1016/j.actpsy.2015.09.005, 26386782

[ref2] Aponte E. A. Tschan D. G. Stephan K. E. Heinzle J. (2018). Inhibition failures and late errors in the antisaccade task: influence of cue delay. J. Neurophysiol. 120, 3001–3016. doi: 10.1152/jn.00240.2018, 30110237

[ref3] Dang J. Barker P. Baumert A. Bentvelzen M. Berkman E. Buchholz N. . (2021). A multilab replication of the ego depletion effect. Soc. Psychol. Personal. Sci. 12, 14–24. doi: 10.1177/1948550619887702 34113424 PMC8186735

[ref4] Dang J. Bjorklund F. Backstrom M. (2017). Self-control depletion impairs goal maintenance: a meta-analysis. Scand. J. Psychol. 58, 284–293. doi: 10.1111/sjop.12371, 28632357

[ref5] Forestier C. de Chanaleilles M. Boisgontier M. P. Chalabaev A. (2022). From ego depletion to self-control fatigue: a review of criticisms along with new perspectives for the investigation and replication of a multicomponent phenomenon. Motiv. Sci. 8, 19–32. doi: 10.1037/mot0000262

[ref6] Frischkorn G. T. Oberauer K. (2025). Is the antisaccade task a valid measure of inhibition? J. Exp. Psychol. Gen. 154, 2456–2481. doi: 10.1037/xge0001808 40569725

[ref7] Lin H. Saunders B. Friese M. Evans N. J. Inzlicht M. (2020). Strong effort manipulations reduce response caution: a preregistered reinvention of the ego-depletion paradigm. Psychol. Sci. 31, 531–547. doi: 10.1177/0956797620904990, 32315259 PMC7238509

[ref8] Lurquin J. H. Miyake A. (2017). Challenges to ego-depletion research go beyond the replication crisis: a need for tackling the conceptual crisis. Front. Psychol. 8:568. doi: 10.3389/fpsyg.2017.00568, 28458647 PMC5394171

[ref9] Mangin T. Audiffren M. Lorcery A. Mirabelli F. Benraiss A. Andre N. (2022). A plausible link between the time-on-task effect and the sequential task effect. Front. Psychol. 13:998393. doi: 10.3389/fpsyg.2022.998393, 36389536 PMC9643466

[ref10] Meier M. E. Smeekens B. A. Silvia P. J. Kwapil T. R. Kane M. J. (2018). Working memory capacity and the antisaccade task: a microanalytic-macroanalytic investigation of individual differences in goal activation and maintenance. J. Exp. Psychol. Learn. Mem. Cogn. 44, 68–84. doi: 10.1037/xlm0000431, 28639800 PMC5741546

[ref11] Pierce J. E. McDowell J. E. (2016). Effects of preparation time and trial type probability on performance of anti- and pro-saccades. Acta Psychol. 164, 188–194. doi: 10.1016/j.actpsy.2016.01.013, 26829023

[ref12] Unsworth N. Robison M. K. Miller A. L. (2022). On the relation between working memory capacity and the antisaccade task. J. Exp. Psychol. Learn. Mem. Cogn. 48, 1420–1447. doi: 10.1037/xlm0001060, 34516206

[ref13] Unsworth N. Schrock J. C. Engle R. W. (2004). Working memory capacity and the antisaccade task: individual differences in voluntary saccade control. J. Exp. Psychol. Learn. Mem. Cogn. 30, 1302–1321. doi: 10.1037/0278-7393.30.6.1302, 15521806

[ref14] Van Gerven P. W. M. Hurks P. P. M. Bovend'Eerdt T. J. H. Adam J. J. (2016). Switch hands! Mapping proactive and reactive cognitive control across the life span. Dev. Psychol. 52, 960–971. doi: 10.1037/dev0000116, 27124653

[ref15] Vohs K. D. Schmeichel B. J. Lohmann S. Gronau Q. F. Finley A. J. Ainsworth S. E. . (2021). A multisite preregistered paradigmatic test of the ego-depletion effect. Psychol. Sci. 32, 1566–1581. doi: 10.1177/0956797621989733, 34520296 PMC12422598

