# Supplementary material for: Prior high demand is associated with lower subsequent performance in a manual antisaccade task
Source: Front Psychol. 2026 Aug 5;17:1882059. doi: 10.3389/fpsyg.2026.1882059 (PMC13486158; doi:10.3389/fpsyg.2026.1882059)
Supplement: Supplementary file 1 [file Data_Sheet_1.PDF]

Supplementary Material

Supplementary Methods

During the formal phase of Task 1, difficulty was adjusted on a trial-by-trial basis according to previous-trial accuracy. The number of blocks ranged from 11 to 17, switching intensity ranged from 1 to 5, and block presentation duration ranged from 0.1 to 0.8 s. After correct responses, difficulty increased; after incorrect responses, difficulty decreased. No trial-by-trial feedback was provided during the formal phase.

Supplementary Figure Captions

Supplementary Figure S1. Overall Task 1 accuracy by group. Error bars represent 95% confidence intervals.

Supplementary Figure S2. Adaptive block-counting task schematic. Participants viewed sequences of small and large blocks. In the low-demand version, participants reported the total number of blocks. In the high-demand version, participants reported the numbers of small and large blocks separately. Difficulty was adjusted during the formal phase based on previous-trial accuracy.

Supplementary Tables

Full supplementary statistical tables are provided in the accompanying Supplementary Tables S1-S16 workbook. The index below gives the table labels used in the revised manuscript and response letter.

Table label	Content
Supplementary Table S1	Group comparability and technical summary
Supplementary Table S2	Accuracy omnibus ANOVA
Supplementary Table S3	Accuracy pooled-error Pro/Anti simple interactions
Supplementary Table S4	Accuracy Anti Group simple-simple main effects
Supplementary Table S5	Accuracy Anti CTI simple-simple main effects
Supplementary Table S6	Accuracy CTI Holm comparisons
Supplementary Table S7	Accuracy descriptives
Supplementary Table S8	Correct-trial RT omnibus ANOVA
Supplementary Table S9	Correct-trial RT pooled-error simple interactions
Supplementary Table S10	Correct-trial RT descriptives and CTI pairwise comparisons
Supplementary Table S11	Recorded OS/platform distribution
Supplementary Table S12	Frame-rate descriptives
Supplementary Table S13	Frame-rate sensitivity model
Supplementary Table S14	Pooled-error, hierarchical-testing, Greenhouse-Geisser, and familywise statistical note

Supplementary Table S15	Recorded-platform subset Accuracy ANOVA
Supplementary Table S16	Recorded-platform subset correct-trial RT ANOVA
